# Attitudes and practices toward COVID‐19 precautionary measures: A comparative study of health professionals and public

**DOI:** 10.1002/brb3.3267

**Published:** 2023-09-27

**Authors:** Muhammad Jawad Hashim, Sarah Jamil, Murriam Masood, Romona Devi Govender, Ali Rashed Al Shamsi, Anwar Al Zaabi, Moien AB Khan, Aqeel Aziz Saleem, Gohar Jamil

**Affiliations:** ^1^ Department of Family Medicine, College of Medicine and Health Sciences United Arab Emirates University Al Ain United Arab Emirates; ^2^ Department of Medicine Tawam Hospital Al Ain United Arab Emirates; ^3^ Department of Medicine Rashid Hospital Dubai United Arab Emirates; ^4^ Cardiology Division Department of Medicine Tawam Hospital Al Ain United Arab Emirates

**Keywords:** attitude, COVID‐19, knowledge, practices, prevention, SARS‐CoV‐2, social precautions

## Abstract

**Background:**

Containment of the COVID‐19 pandemic has been impaired by the denial and defiance of preventive recommendations.

**Aims:**

We aimed to study the attitudes toward COVID‐19 social measures among laypersons and healthcare professionals.

**Methods:**

We conducted a cross‐sectional study in the United Arab Emirates using a self‐administered online questionnaire. Both healthcare workers and laypersons were actively recruited. In addition to sociodemographic variables, the questionnaire included questions on anxiety, knowledge, and defiance related to COVID‐19.

**Results:**

A total of 615 individuals with a mean age of 32 years (SD, 12) participated. Females comprised 69% and healthcare workers constituted 60% of the study sample. Among laypersons, over 42% reported having social gatherings at home, and 44% admitted to visiting crowded places. More than half of the respondents felt increased anxiety. Previous COVID‐19 infection did not affect attitudes or anxiety levels. Knowledge about COVID‐19 was higher among those who were more educated (*r* = .21). Healthcare workers had lower anxiety than laypersons (*p* = .002). COVID‐19 anxiety was higher among older persons and did not decrease with more knowledge. COVID‐19 defiance was higher among younger male respondents from larger households and did not correlate with knowledge. Multivariate analysis showed more defiant attitudes at younger ages.

**Conclusions:**

Anxiety‐related to the COVID‐19 pandemic is more common in older individuals, whereas younger persons were more likely to deny and defy prevention recommendations despite having knowledge of viral transmission. Voluntary compliance by young individuals requires an engaging communication strategy to generate more compassionate attitudes.

## Graphical abstract

We aimed to study the attitudes toward COVID‐19 social measures among laypersons and healthcare professionals, as the containment of the COVID‐19 pandemic has been impaired by denial and defiance of preventive recommendations. We conducted a cross‐sectional study in the United Arab Emirates using a self‐administered online questionnaire and found that anxiety related to the COVID‐19 pandemic is more common in older individuals, whereas younger persons were more likely to deny and defy prevention recommendations despite having knowledge of virus transmission.

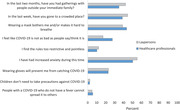

## 1 INTRODUCTION

Pandemics impact mental health which in turn affects social behavior and compliance (Amerio et al., 2021; Khan et al., 2021; Petzold et al., 2020). Social isolation can worsen mental well‐being by reducing access to psychiatric care (Ambrosetti et al., 2021). The impact of pandemics can be felt across the society including on women and children (García‐Fernández et al., 2021; Nobari et al., 2021). Control of pandemics requires voluntary social cooperation (Leung et al., 2022). People need to follow social precautions such as wearing face masks and staying at home during outbreaks. Unfortunately, compliance with recommended measures remained low during the recent pandemic (Leung et al., 2022). Attitudes and behaviors toward public health measures varied across and within communities.

Although social preventive measures were implemented in the interest of public health and safety, at an individual level, the time‐dependent reactions were not always positively accepted and understood (Hassan et al., 2021; Khan et al., 2022; Tsenoli et al., 2021). Some believed that the restrictions were an infringement on civil liberties, others appeared to actively make decisions to engage in behaviors that they considered to be in their personal interests regardless of social implications (Denford et al., 2021). Defiance and denial of pandemic preventive measures remain a concern worldwide. The perceived efficacy of these measures together with the fear of the virus still needs to be better understood (Latkin et al., 2021; Magarini et al., 2021). There is a paucity of research studies on the predictors of denial and defiance of social precautions.

We studied perceptions and attitudes toward social precautionary measures during the pandemic. We aimed to compare laypersons with healthcare workers in terms of their practices. These factors will be important in controlling future pandemics.

## 2 MATERIALS AND METHODS

### 2.1 Study design and population

This cross‐sectional study was conducted after being approved by the Institutional Review Board from the Abu Dhabi, Department of Health (DOH/CVDC/2020/2018). The study was conducted as per the Helsinki Declaration protecting human subjects in research (World Medical Association, 2013). We have followed the Strengthening the Reporting of Observational Studies in Epidemiology (Appendix 1) statement to report this study (Elm et al., 2007). Data was collected between 1 October 2020 and 31 May 2021. The survey was collected using convenience and snowball sampling techniques. Survey responses were collected anonymously. Prior to the study, each of the study participants consented by agreeing to voluntary participation information. Participants were recruited from a major tertiary hospital and a primary healthcare center. The target population included healthcare professionals and public residing in the United Arab Emirates (UAE). Participants who were aged 18 or more, understood the content of the study, and who agreed to participate in the study were allowed to complete the web‐based questionnaire. Healthcare professionals included physicians, nurses, pharmacists, dentists, and other allied health professionals.

### 2.2 Study questionnaire design

The questionnaire was developed by the researchers based upon instruments validated previous studies (Alhazmi et al., 2020; Zhang et al., 2020; Zhong et al., 2020). The study utilized a 31‐item self‐administered, bilingual (English, Arabic) web‐based questionnaire. The survey questionnaire was initially developed in English and then forward translated and subsequently back translated using best practices for translation and cultural adaptation (Wild et al., 2005). The survey was distributed via email and various social media platforms. Data collection was conducted using Google Forms. A pilot study of 10 healthcare professionals and 10 laypeople living in the UAE was conducted to determine the face validity and usability of the study tool. The questionnaire took around 5–10 min to complete and consisted of multiple choice, true and false, and fill in the blank items. As a result of piloting feedback, the study tool was revised to ensure that it was easily understood and completed.

The questionnaire had four sections as follows: demographics (17 questions), knowledge (20 questions), attitude (22 questions), and practices (12 questions). Based on mean responses to relevant items in the questionnaire, scores were calculated for knowledge (questionnaire items in the “knowledge” section: 1–16), anxiety (questionnaire items in the “attitude” section: 1, 2, 4 [negatively coded], 16, and 17), and defiance (questionnaire items in the “attitude” section: 7, 8, and 11, as well as items in the “practices” section: 1 [negatively coded], 2, and 5) related to the COVID‐19 pandemic.

The three primary outcomes were the scores on knowledge, anxiety, and defiance. Predictors (independent) variables included age, sex, national origin, level of education, occupation (healthcare vs. non‐healthcare), household size, and previous diagnosis of COVID‐19. An estimated sample size of 402 participants was required based on approximately 50% correct responses in the knowledge score and a 95% confidence interval width of 10% (binomial calculation).

### 2.3 Statistical analysis

Descriptive statistics were used to summarize participant responses to individual items as well as the knowledge, anxiety, and defiance scores. Bivariate analysis included Pearson correlation coefficients and one‐way ANOVA tests. Multivariate analysis was conducted using partial least squares regression (with the maximum number of latent factors set to 5). All statistical analyses were conducted using SPSS version 28 (IBM SPSS Inc., 2021). An alpha level of *p* < .05 was considered statistically significant, and missing values were less than 1% and were not imputed.

## 3 RESULTS

The study had 615 participants with a broad range of age groups and ethnic backgrounds (Table 1). Females constituted 69% (422 out of 615) of the sample. Healthcare care professionals comprised 60% (369), which included physicians (70), nurses (137), pharmacists (9), as well as dentists, lab technicians, midwives, phlebotomists, physiotherapists, and respiratory therapists. Unemployed status was reported by 5.5% (27) of the respondents, with an additional 0.8% (5) individuals reporting unemployment due to the economic impact of the COVID‐19 pandemic. The study sample also included 19 homemakers (3.1%) and 193 students (31.4%). All respondents were living in the UAE, with the majority residing in Abu Dhabi and Dubai. National origin ranged from South Asia (India, Pakistan, and Bangladesh) and Arab countries (UAE, Egypt, Jordan, Syria, Sudan, and Algeria) to Western origin (United States, United Kingdom, and Australia) and East Asia (Philippines and Malaysia) as well as others such as South Africa, Turkey, and Iran. Among the participants, 44 (7.2%) reported that they had been infected in the past with SARS‐CoV‐2. The rate of past infection was higher among healthcare professionals.

**TABLE 1:** Knowledge, attitudes, and practices toward COVID‐19.

*Characteristic*	*Health professionals (n = 369)*	*Laypersons (n = 246)*	*Total (n = 615)*	*p‐Value*
	*N* (%)	*N* (%)	*N* (%)	
Age (years)				.003
24 or younger	128 (34.7)	100 (40.7)	228 (37.1)	
25–49	190 (51.5)	130 (52.8)	320 (52.0)	
50–64	51 (13.8)	14 (5.7)	65 (10.6)	
64 or older	0	2 (0.8)	2 (0.3)	
Gender				.002
Females	271 (73.4)	95 (38.6)	193 (31.4)	
Males	98 (26.6)	151 (61.4)	422 (68.6)	
Education				<.001
Primary	1 (0.3)	4 (1.6)	5 (0.8)	
Secondary	1 (0.3)	2 (0.8)	3 (0.5)	
High school	18 (4.9)	54 (22.0)	72 (11.7)	
University	349 (94.6)	186 (75.6)	535 (87.0)	
Regional origin				<.001
South Asian	132 (35.8)	90 (36.6)	222 (36.1)	
Arab	107 (29.0)	67 (27.2)	174 (28.3)	
Western	30 (8.1)	43 (17.5)	73 (11.9)	
East Asian	79 (21.4)	10 (4.1)	89 (14.5)	
Other	21 (5.7)	36 (14.6)	57 (9.3)	
Household size				.58
1	58 (15.7)	44 (18.4)	102 (16.8)	
2 to 5	235 (63.7)	141 (59.0)	376 (61.8)	
6 to 9	63 (17.1)	42 (17.6)	105 (17.3)	
10 or more	13 (3.5)	12 (5.0)	25 (4.1)	
COVID‐19 diagnosed	29 (7.9)	15 (6.1)	44 (7.2)	.41

**FIGURE 1:**
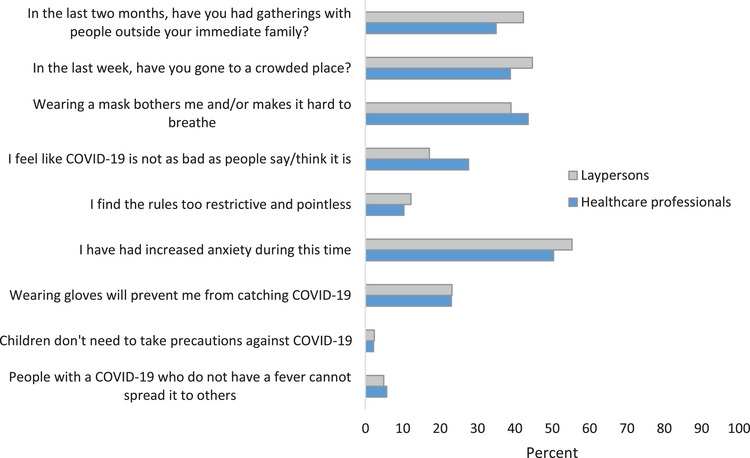
Laypersons and health professionals’ responses to selected questions (*n* = 615).

**FIGURE 2:**
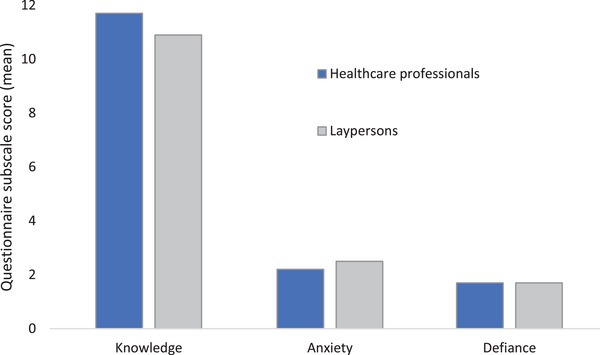
Comparison of healthcare professionals versus laypersons for COVID knowledge, anxiety, and defiance subscales.

**FIGURE 3:**
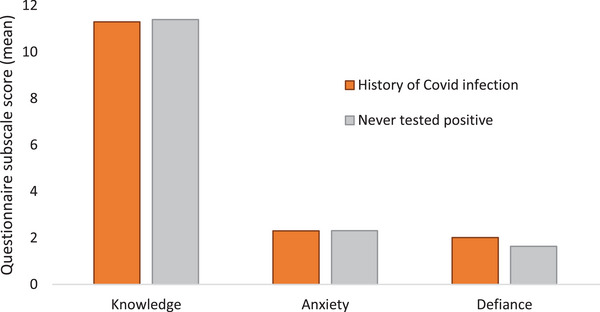
Comparison of individuals with a history of COVID‐19 versus those who never had the infection. *Note*: None of the differences between the two groups were statistically significant (*p* > .05; one‐way ANOVA).

Responses from participants revealed evidence of gaps in knowledge, the defiance of social protective measures, and COVID‐19‐related anxiety (Figure 1). Over 40% of the participants reported having social gatherings at home and going to crowded places. Healthcare professionals had greater knowledge and less anxiety related to the pandemic (Figure 2). However, Defiance scores were similar across public and healthcare workers. Comparison of those with a history of SAR‐CoV‐2 infection with those who never had a positive result showed no differences in knowledge, anxiety, or defiance scores (Figure 3).

Social media influence was present to a small extent. About 17% (104 out of 615 respondents) admitted following COVID‐19 advice of nonmedical persons on social media.

### 3.1 Bivariate associations

COVID‐19 knowledge score did not correlate with the Anxiety score (*r* = −.02) or the defiance score (*r* = −.07). Anxiety and defiance scores did not correlate with each other (*r* = .08). As expected, those with greater educational attainment tended to have higher COVID‐19 knowledge scores (*r* = .21; 95% CI, .13, .28). Larger household size was not associated with higher anxiety scores (*r* = .06). Participants’ age correlated weakly with knowledge score (*r* = .15), anxiety score (*r* = −.14), or defiance score (*r* = −.14).

Male respondents had greater COVID‐19 defiance scores than females (mean score 1.82 vs. 1.59 among females; one‐way ANOVA, *p* = .036). Females had a slightly higher COVID‐19 Anxiety score; however, this was not statistically significant (mean score 2.38 vs. 2.16 among males; one‐way ANOVA, *p* = .08). Knowledge scores were similar in both sexes (11.5 vs. 11.4; *p* = .26).

Comparison of healthcare workers with laypersons revealed higher knowledge scores among professionals (11.7 vs. 10.9; *p* < .001). On the other hand, the COVID‐19 anxiety scores were greater among laypersons (2.53 vs. 2.16; *p* = .002). COVID‐19 defiance scores were similar across both groups (1.6 vs. 1.3; *p* = .78). Defiance scores were somewhat higher among those had a history of positive COVID test (mean score 2.02) than those who did not (1.64), but this was not statistically significant (*p* = .071).

### 3.2 Multivariate analysis

On partial least squares regression, knowledge about COVID‐19 was predicted by greater education and being a healthcare worker and to a lesser extent by smaller household size, but not by the age of the respondent. COVID‐19 anxiety score was predicted by female gender and being a non‐healthcare worker (layperson) but not by other variables such as age, education, or household size. Similarly, the COVID‐19 defiance score was predicted by male gender and to less extent by lower education lower age but not by other variables collected in the study.

## 4 DISCUSSION

A key finding emerging from this study is the high self‐reported defiance of COVID‐19 precautionary measures among younger male individuals. Knowledge about the virus did not correlate with lower anxiety related to the pandemic. Remarkably, COVID‐19 denial and defiance were equally prevalent among health professionals and laypersons.

In our study, a substantial proportion of participants continued attending social gatherings and visiting crowded places. Alarmingly, many respondents indicated agreement with statements such as the pandemic is overblown, the rules are pointless and too restrictive, and the COVID‐19 is not as bad as people say it is. These findings are concerning. COVID‐19 skepticism has also been reported in an online questionnaire survey of 683 respondents from the United States (Latkin et al., 2021). The US survey found an association of skepticism with younger age, better health, and politically conservative views. However, despite public health measures such as social distancing, facemasks, and travel restrictions, the spread of SARS‐CoV‐2 infection worldwide has continued. This is partly due to unfavorable attitudes and practices toward these precautionary measures.

Furthermore, skepticism correlated with the lower use of preventive measures such as wearing a mask. A survey of 681 individuals in the United Kingdom revealed that 92.8% did not adhere to the social distancing rules; although age, gender, ethnicity, education, and employment status were not predictive (Hills & Eraso, 2021). In comparison, in our study, COVID‐19 defiance was associated with lower age but not with educational attainment, health professional status, or knowledge of the disease itself. This implies that public health education needs to target younger individuals in this region. Younger adults need more focused educational efforts to address irrational beliefs and defiant attitudes toward social measures. Building trust in the community requires using a variety of dissemination channels as well as the use of reliable information (Fridman et al., 2020). As in our study results, unverified news sources such as social media can lead to the defiance of preventive recommendations (Ghaddar et al., 2022).

The level of anxiety was high in our study sample. This is consistent with other research studies evaluating the psychological effects of regional lockdowns and social restrictions (Ammar et al., 2020). An earlier study found high levels of emotional exhaustion, irritability, and sleep disturbances (Cheikh Ismail et al., 2020). We found a mildly lower level of anxiety among health professionals compared to laypersons. This is possibly due to a higher level of knowledge of the disease and its prognosis among healthcare workers.

It is concerning to note that younger individuals, both layperson and healthcare professionals, regardless of their educational attainment and prior knowledge of the virus, are more likely to deny and defy protective measures. An online survey of adults living in North America and Europe also showed that men and younger individuals showed lower adherence (Coroiu et al., 2020). In one region, almost 30% of individuals did not believe in the use of facemasks (Meo et al., 2021). An in‐depth review of irrational beliefs about COVID‐19 has shown that misinformation tends to cluster with particular socio‐environmental factors such as lower education, younger age, low levels of trust, avoidance of uncertainty, collective narcissism, and a conspiracy‐prone mindset (Magarini et al., 2021). Although legally enforced travel restrictions and mandatory testing are needed to ensure compliance, public health campaigns should highlight compassionate attitudes toward social measures. Voluntary self‐isolation by infected persons is critical to control the spread. Yet, young individuals with mild symptoms cannot be coerced to stay at home. Voluntary self‐isolation can only occur if they harbor compassionate attitudes toward the safety of others. Social support has been found to be associated with lower risk of depression in a recent cross‐sectional survey (Grey et al., 2020). In a study from UAE, divorced mothers were more likely to be hesitant about vaccinations for their children (Alsuwaidi et al., 2020). Hence, contextual factors such as family and community may play a role in this illness (Hashim, 2016). As supportive care remains the mainstay of treatment, patient‐centered communication plays a central role in allaying COVID‐19 anxiety (Hashim, 2017).

To our knowledge, this is the first study conducted in the UAE evaluating defiance of public health measures Minor grammatical edits made. Our study highlights the need for holistic measures and policies and interaction with the public to modify health behaviors.

There are some limitations inherent in our study design. The study inferences may be limited by the use of an online data collection format; however, the anonymous nature of this medium may foster more candid responses than in‐person interviews. Recall bias can affect participants’ responses. Another limitation of our study was that the sample was over‐representative of university educated individuals, perhaps due to the format of the study. This may affect generalizability of findings. Due to the rapidly evolving nature of the pandemic, a validated questionnaire could not be applied. The cross‐sectional study design does not permit making firm inferences due to the lack of follow‐up over time.

## 5 CONCLUSION

This study highlights the importance of communication strategies for future pandemics. Lack of compliance with preventive measures and unfavorable attitudes were remarkably common. Denial and defiance especially among younger adults can undermine control measures to prevent viral transmission.

Communication strategies should build trust among the lay public and healthcare professionals. Our study underscores the importance of reaching out to younger individuals, building trust, and allaying anxiety about the pandemic. Public health campaigns should focus on connecting with young adults and engaging in meaningful dialog that is congruent to their perspectives. Strict lockdown directives may not be effective if trust is not established. Emotional support is critical to allay anxiety during pandemics.

## AUTHOR CONTRIBUTIONS

All the authors have made substantive contributions to the article and assume full responsibility for its content.

## CONFLICT OF INTEREST STATEMENT

The author(s) declared no potential conflicts of interest with respect to the research, authorship, and/or publication of this article.

### Peer Review

The peer review history for this article is available at https://publons.com/publon/10.1002/brb3.3267.

## Data Availability

The data that support the findings of this study are available from the corresponding author upon reasonable request.

## APPENDIX 1

### 1.1

STROBE statement—checklist of items that should be included in reports of observational studies

Attitudes and practices toward COVID‐19 precautionary measures: a comparative study of health professionals and public

**Table:**

	Item no.	Recommendation	Page no.	Relevant text from manuscript
**Title and abstract**	1	(a) Indicate the study's design with a commonly used term in the title or the abstract	1	Abstract
		(b) Provide in the abstract an informative and balanced summary of what was done and what was found	1	Yes
Introduction				
Background/rationale	2	Explain the scientific background and rationale for the investigation being reported	4	Yes
Objectives	3	State specific objectives, including any prespecified hypotheses	4	Yes
Methods				
Study design	4	Present key elements of study design early in the paper	4	Yes
Setting	5	Describe the setting, locations, and relevant dates, including periods of recruitment, exposure, follow‐up, and data collection	4	Yes
Participants	6	*Cross‐sectional study*—give the eligibility criteria, and the sources and methods of selection of participants	4	Yes
		(b) *Cohort study*—for matched studies, give matching criteria and number of exposed and unexposed		
*Case–control study*—for matched studies, give matching criteria and the number of controls per case				
Variables	7	Clearly define all outcomes, exposures, predictors, potential confounders, and effect modifiers. Give diagnostic criteria, if applicable	5	Yes
Data sources/measurement	8*	For each variable of interest, give sources of data and details of methods of assessment (measurement). Describe comparability of assessment methods if there is more than one group	6–7	*Yes*
Bias	9	Describe any efforts to address potential sources of bias	8	Yes
Study size	10	Explain how the study size was arrived at	5	Yes
Quantitative variables	11	Explain how quantitative variables were handled in the analyses. If applicable, describe which groupings were chosen and why	5–8	Yes
Statistical methods	12	(a) Describe all statistical methods, including those used to control for confounding	6	Yes
		(b) Describe any methods used to examine subgroups and interactions		
		(c) Explain how missing data were addressed		
		(d) *Cohort study*—if applicable, explain how loss to follow‐up was addressed		
*Case–control study*—if applicable, explain how the matching of cases and controls was addressed				
*Cross‐sectional study*—if applicable, describe analytical methods taking account of sampling strategy	N/A			
		(e) Describe any sensitivity analyses		
Results				
Participants	13*	(a) Report numbers of individuals at each stage of study—e.g., numbers potentially eligible, examined for eligibility, confirmed eligible, included in the study, completing follow‐up, and analyzed	N/A as conveyance sampling as per eligibility criteria	
		(b) Give reasons for nonparticipation at each stage		
		(c) Consider use of a flow diagram		
Descriptive data	14*	(a) Give characteristics of study participants (e.g., demographic, clinical, and social) and information on exposures and potential confounders	6, Table 1	Yes
		(b) Indicate number of participants with missing data for each variable of interest		
		(c) *Cohort study*—summarize follow‐up time (e.g., average and total amount)		
Outcome data	15*	*Cohort study*—report numbers of outcome events or summary measures over time		
		*Case–control study—*report numbers in each exposure category, or summary measures of exposure		
		*Cross‐sectional study—*report numbers of outcome events or summary measures	*6–8*	*Yes*
Main results	16	(a) Give unadjusted estimates and, if applicable, confounder‐adjusted estimates and their precision (e.g., 95% confidence interval). Make clear which confounders were adjusted for and why they were included	6–8	Yes
		(b) Report category boundaries when continuous variables were categorized		
		(c) If relevant, consider translating estimates of relative risk into absolute risk for a meaningful time period		
Other analyses	17	Report other analyses done—e.g., analyses of subgroups and interactions, and sensitivity analyses		
Discussion				
Key results	18	Summarize key results with reference to study objectives	8	
Limitations	19	Discuss limitations of the study, taking into account sources of potential bias or imprecision. Discuss both direction and magnitude of any potential bias	15	
Interpretation	20	Give a cautious overall interpretation of results considering objectives, limitations, multiplicity of analyses, results from similar studies, and other relevant evidence	9–10	
Generalizability	21	Discuss the generalizability (external validity) of the study results	10	
Funding	22	Give the source of funding and the role of the funders for the present study and, if applicable, for the original study on which the present article is based	11	
